# The Role of the Glass Ceiling Syndrome in Female Healthcare Workers and Its Association With Telogen Effluvium

**DOI:** 10.1111/jocd.70447

**Published:** 2025-10-13

**Authors:** Delal Aydın, Ömer Karakoyun, Erhan Ayhan

**Affiliations:** ^1^ Department of Health Management Faculty of Economics and Administrative Sciences, Fırat University Elazığ Turkey; ^2^ Department of Dermatology and Venereology, Faculty of Medicine Dicle University Diyarbakır Turkey

**Keywords:** glass ceiling, health worker, stress, telogen effluvium, woman

## Abstract

**Background‐Objective:**

Telogen effluvium (TE) type hair loss is the most common cause of diffuse and non‐scarring alopecia in women and may develop on the basis of many etiological causes. Glass ceiling syndrome, which is a popular research topic especially in recent years, is one of the syndromes that define the invisible barriers that women face in their careers and can cause long‐term stress during periods of exposure. Women working in the health sector are frequently exposed to the glass ceiling, and this situation has negative effects on skin health. The aim of the study is to investigate the relationship between glass ceiling syndrome‐induced stress and telogen effluvium, which has not been examined before. In addition, another aim of the study is to determine the effect of socio‐demographic variables such as age, gender, and education level on stress symptoms telogen effluvium.

**Materials and Methods:**

This study was conducted on 108 female participants working in University Hospitals. Questionnaires were used to determine how the participants experienced the glass ceiling syndrome, their professional and marital status, and their level of education. In addition, an examination including a pull test and trichoscopy for telogen effluvium disease was performed by specialized doctors.

**Results:**

56 (51.85%) of 108 female health workers were diagnosed with telogen effluvium. It was determined that 73 (67.59%) of them had glass ceiling experience. Of the 108 female health workers, 36 were doctors, 44 were nurses, and 28 were other health workers. Regarding their education levels, 36 of them have a master's degree, 18 have a bachelor's degree, 34 have an associate's degree, and 20 have a high school degree. As for marital status, 71 of them were married, 24 of them were single, and 13 of them were divorced. A significant statistical relationship was found between telogen effluvium and glass ceiling syndrome and education and occupational status. At the same time, a significant statistical relationship was found between glass ceiling experience and education and occupational status.

**Conclusion:**

Glass ceiling syndrome is statistically significantly associated with telogen effluvium in female health workers, and it is emphasized that this situation should not be neglected as a source of intense stress during treatment. In addition, professional and marital status and education levels are also seen to feed this intense stress source.

## 1 Introduction

Glass ceiling syndrome has been in the literature for a long time as a metaphor expressing the invisible barriers that women face in business life [1].

It was first defined by Hymowitz and Schelhardt in an article published in the Wall Street Journal in 1986. In this article, the seriousness of the obstacles faced by women in accessing senior management positions was emphasized, and the prejudices faced by women in the workplace were discussed. Hymowitz and Schelhardt defined the glass ceiling concept as a career barrier for women and drew attention to the pressure this barrier creates on female employees [2].

Glass ceiling syndrome is also defined as invisible barriers that limit the career development of employees based on gender and other social factors in the workplace [3].

It has been reported that especially female employees in the health sector have difficulty advancing in their careers due to glass ceiling syndrome, and this situation leads to long‐term stress [4].

Pressure and stress applied to women negatively affect their performance in business life and may lead to serious consequences on their physical health in the long term [5]. The negative effects of stress on skin health have become an increasingly serious problem for female employees [6]. One of the effects of stress on physical health is skin diseases [7]. It has been reported that many dermatological diseases, especially eczema and acne, are associated with stress and are more common in individuals working under intense workload and emotional pressure [8].

Studies investigating the effects of workplace stress on skin health have mostly focused on female employees. Shively and Clarkson reported that prolonged stress in work life led to hormonal imbalances in women and this negatively affected skin health [9]. In addition, Smith and Jones conducted a large‐scale study showing that the stress that female employees are exposed to at work may cause dermatological disorders [10]. These findings also indicate that stress coping strategies for female employees should be developed.

It is known that stress disrupts the skin barrier by weakening the natural defense mechanisms of the skin and may cause inflammation [11]. Many stress hormones, especially cortisol, play an important role in this biological process. It has been reported that high levels of cortisol can lead to the emergence of various dermatological disorders manifested by symptoms such as skin dryness, itching, and inflammation [12]. Women are under constant stress due to discrimination and glass ceiling pressures they face in business life, and this is thought to negatively affect their skin health.

Telogen effluvium (TE) is one of the most common causes of hair loss and usually occurs when hair follicles leave the normal growth cycle and enter the resting (telogen) phase. In telogen effluvium, due to a trigger such as stress, a large portion of the hair follicles leave the anagen phase early and enter the telogen phase [13]. Constant stress at the workplace increases the level of stress hormones such as cortisol. High cortisol levels may affect hair follicles and lead to hair loss [14]. Chronic stress also disrupts the hair growth cycle, causing more hair to enter the telogen phase. Stress can contribute to inflammation of hair follicles and hair loss by suppressing the immune system [15]. Increased adrenaline and other stress hormones due to stress at work can reduce blood flow to the scalp. This can prevent adequate oxygen and nutrient transport to the hair follicles and increase hair loss [14]. Stress at the workplace can also disrupt sleep patterns and has been reported to lead to unhealthy eating habits [16]. Lack of sleep and malnutrition also affect the health of hair follicles and may trigger hair loss [17, 18]. Conditions such as intensity, pressure, or mobbing in the workplace may lead to emotional wear and tear in individuals. This may make hair loss more prominent among the physical symptoms of stress.

This study aims to examine the relationship between glass ceiling syndrome experiences of female healthcare workers and telogen effluvium. The questionnaire used in the study includes questions to assess healthcare workers' attitudes towards their careers and emotional states, as well as questions to determine the effects of stress on physical health. The findings of the study are expected to draw attention to the importance of policies that support gender equality in the workplace and provide guidance on protecting the health of employees.

## 2 Methods

The population of the study consisted of female healthcare professionals working at University Hospital. A total of 108 participants were recruited using a convenience sampling method, and the data obtained were evaluated. Both qualitative and quantitative research methods were employed to explore stress‐related health problems caused by the glass ceiling syndrome.

### 2.1 Sampling Procedure and Questionnaire Distribution

According to the *2024 Annual Administrative Activity Report* of the University, there were 523 female academic staff and 935 female healthcare personnel, totaling approximately 1458 female healthcare workers employed at the university hospitals [19]. Using a convenience sampling method, 130 self‐administered questionnaires were distributed in person by trained research assistants during working hours between April and May 2025. Of these, 108 were fully completed and included in the final analysis. After completing the survey, all participants were referred to the dermatology department for clinical examination and laboratory testing to confirm telogen effluvium.

### 2.2 Inclusion and Exclusion Criteria

The inclusion criteria were: female healthcare professionals aged between 22 and 60; at least 1 year of employment at University Hospital; voluntary participation with signed informed consent; and availability for clinical dermatological examination.

The exclusion criteria included: diagnosed dermatological conditions other than TE (e.g., alopecia areata, psoriasis); recent childbirth (within the past 6 months), surgery, or significant physical trauma; use of medications known to impact the hair cycle (e.g., isotretinoin, chemotherapy); and abnormal laboratory values indicating alternative causes such as iron‐deficiency anemia or thyroid dysfunction.

A structured General Health and Lifestyle Form was also used to screen for potential confounding factors such as chronic illness, psychiatric conditions, medication use, sleep patterns, dietary habits, recent emotional trauma, and family‐ or work‐related stressors. In addition, participants underwent laboratory tests including complete blood count (CBC), serum ferritin, and thyroid‐stimulating hormone (TSH) levels. Those with abnormal findings or alternative causes of hair loss were excluded.

### 2.3 Mental Health Evaluation

Mental health was indirectly assessed using the Career Pathway Survey (CPS), which includes emotional and cognitive subdimensions such as Resignation and Perceived Control. Example items include:

- “Sometimes I feel like my job is beyond my control”
- “I feel I am blocked from promotion despite being qualified”

Additionally, during dermatological consultation, a brief stress symptom checklist was administered based on DSM‐5 criteria (sleep disturbances, fatigue, low mood, irritability). While no formal psychiatric scale (e.g., GHQ, BDI) was employed, participants showing prominent symptoms were advised to seek further evaluation.

### 2.4 Glass Ceiling Syndrome Scale

The Career Pathway Survey (CPS), developed by Smith et al. (2012), was used to assess participants' perceptions of the glass ceiling [20]. The scale also allowed examination of differences based on age, education, and marital status. The CPS is a validated 38‐item instrument encompassing four dimensions: denial, resilience, resignation, and acceptance. The Turkish version of the CPS, adapted and validated by Sarioğlu (2022), demonstrated high internal consistency (Cronbach's *α* = 0.89) [21]. Prior to full administration, a pilot test was conducted with 15 female healthcare workers to ensure linguistic clarity and cultural relevance.

Sociodemographic information such as marital status and education level was also collected. Diagnosis of telogen effluvium was confirmed through a dermatological assessment including a pull test and trichoscopy, performed by board‐certified dermatologists.

### 2.5 Statistical Analysis

Statistical analyses were performed using SPSS version 21.0. Descriptive statistics included means, medians, standard deviations, frequencies, and incidence rates. The Kolmogorov–Smirnov test, paired *t*‐test, Wilcoxon test, Pearson Chi‐square (*χ* ^2^) test, Yates' correction Chi‐square test, Fisher's exact test, McNemar test, Pearson/Spearman correlation analysis, and logistic regression analysis were used as appropriate. A *p*‐value of < 0.05 was considered statistically significant.

## 3 Findings

**GRAPH 1:**
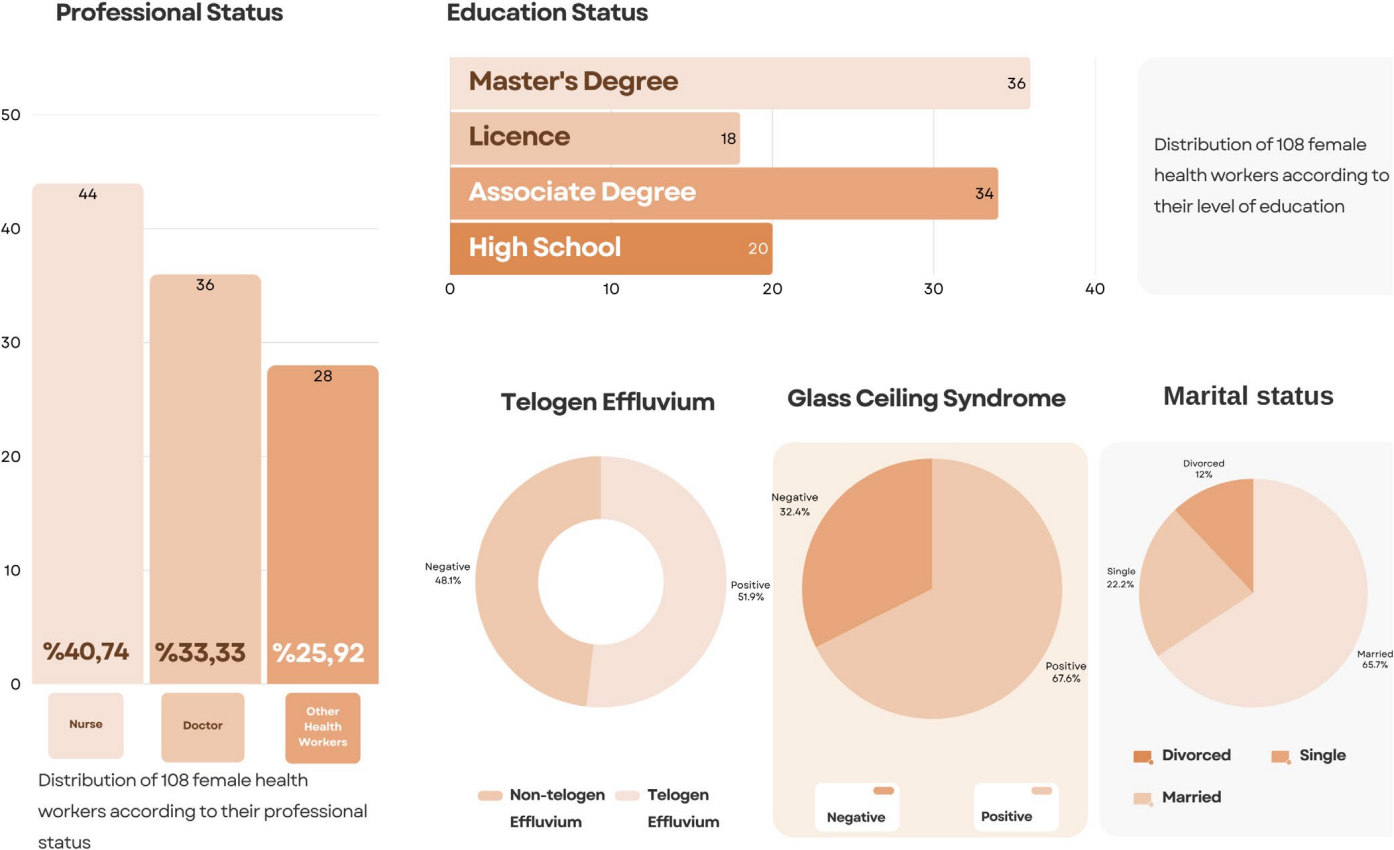
Distribution of 108 female health workers according to research criteria.

**GRAPH 2:**
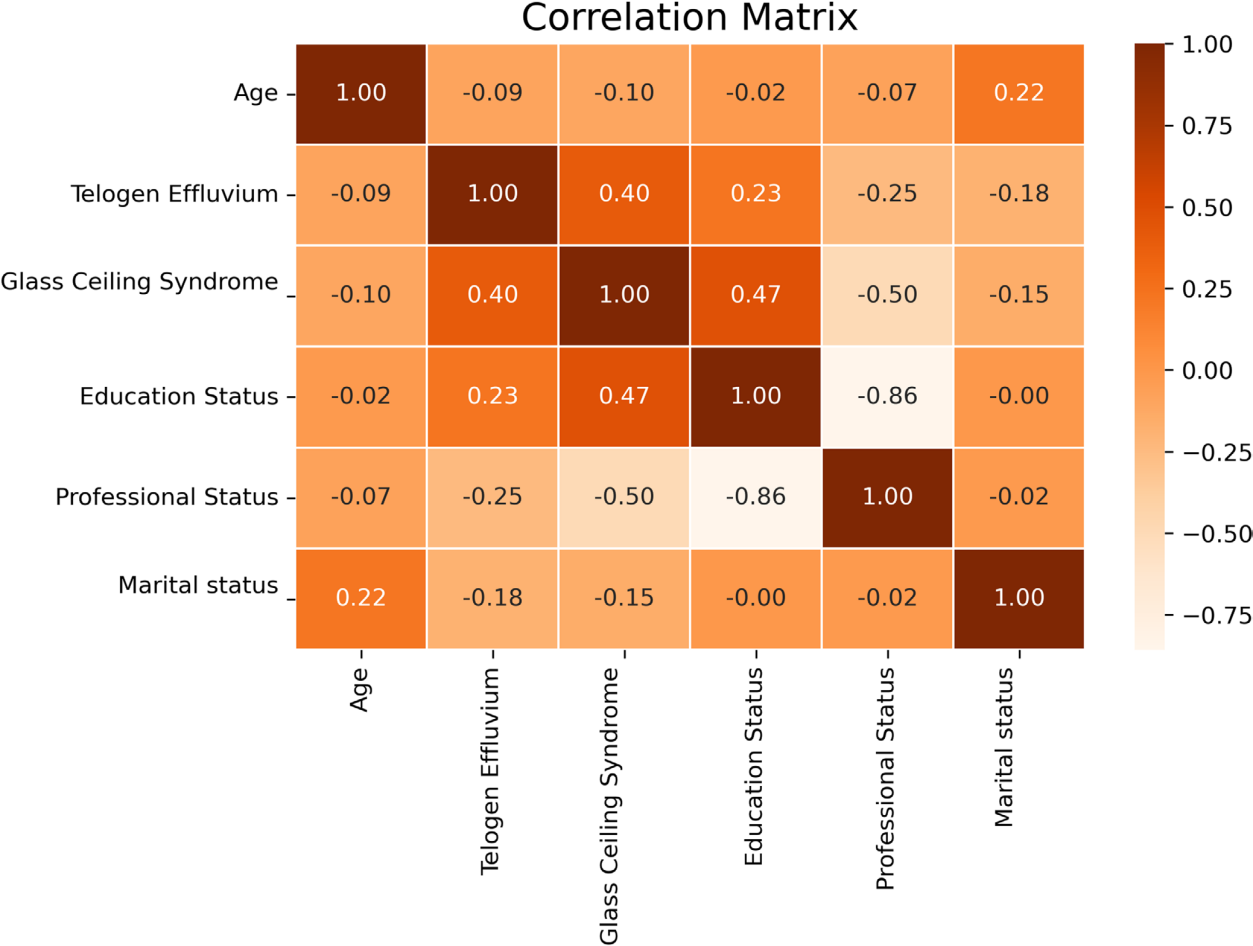
Correlation matrix of the collected data.

**GRAPH 3:**
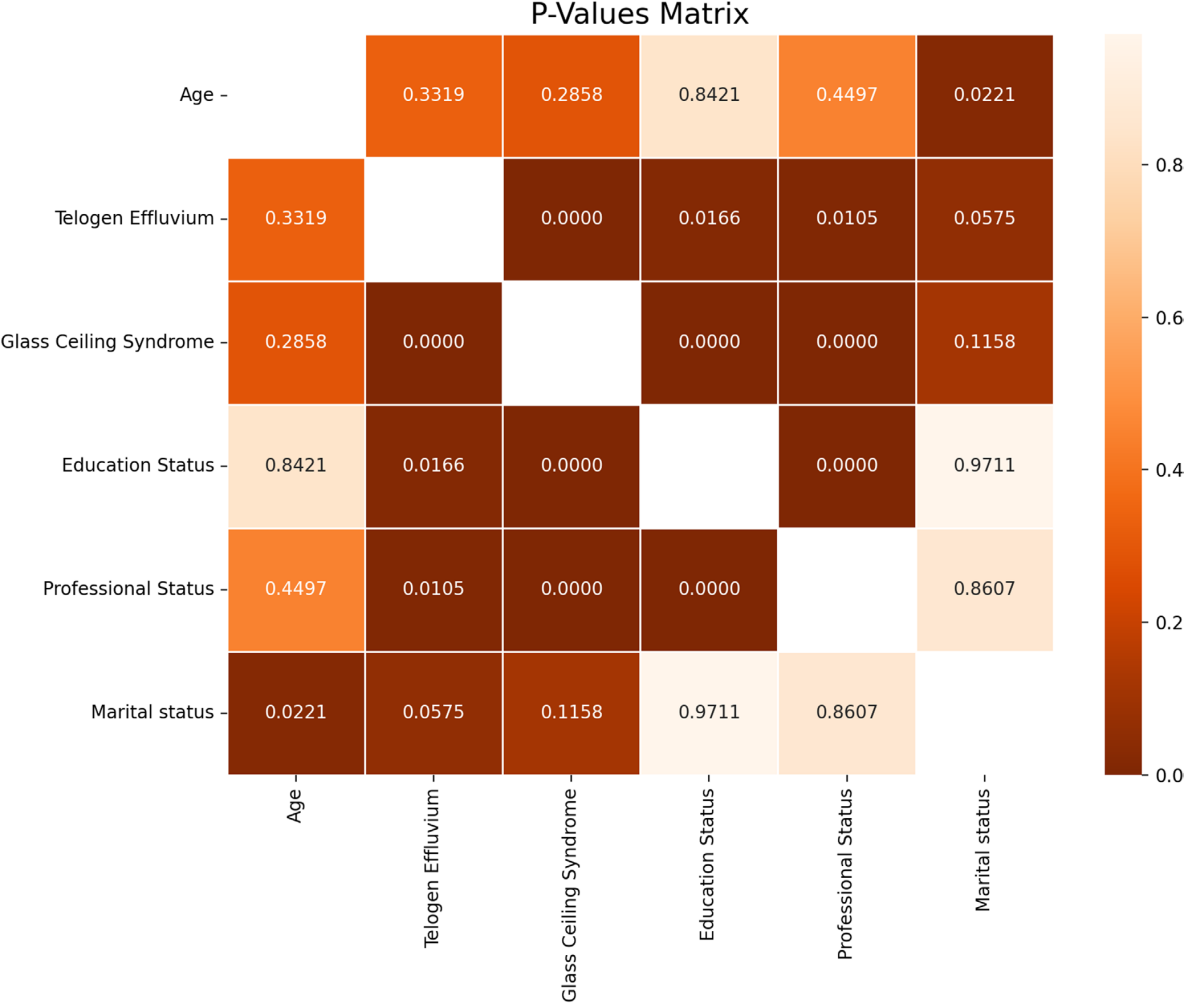
Distribution of statistically significant relationships among data.

56 (51.85%) of 108 female health workers were diagnosed with telogen effluvium. It was determined that 73 (67.59%) of them had glass ceiling experience. Of the 108 female health workers, 36 were doctors, 44 were nurses, and 28 were other health workers. Regarding their education levels, 36 of them have a master's degree, 18 have a bachelor's degree, 34 have an associate's degree, and 20 have a high school degree. As for their marital status, 71 of them are married, 24 of them are single, and 13 of them are divorced. This information can be seen in a more detailed way in Graph 1. A significant statistical relationship was found between telogen effluvium and glass ceiling experience. At the same time, a significant statistical relationship was found between glass ceiling experience and TE and occupational and educational status. Statistical analyses of these data are shown in more detail in Graphs 2 and 3.

## 4 Discussion

Telogen effluvium (TE) type hair loss is a common disease and it is the most common cause of diffuse and non‐scarring alopecia in women and may develop on the basis of many etiological causes [22]. When the normal hair cycle is analyzed, it is known that it consists of 3 periods including anagen, catagen, and telogen. TE, which may be caused by various etiological factors, is an abnormal condition of this hair cycle and many hair follicles in the anagen phase pass prematurely to the telogen phase, resulting in excessive telogen hair loss [23]. While 100 hair losses per day are considered normal in a normal person, this can reach up to 400 in patients with TE. Considering whether this process lasts longer or shorter than 6 months, it can be classified as acute and chronic TE [24]. While etiological reasons can be found in acute telogen effluvium, this is not clear in chronic TE. Many etiological reasons, including normal human physiology such as pregnancy, drugs, severe infections, thyroid dysfunction, anemia, trauma, emotional, and physiological stress, can be seen [25].

Glass ceiling syndrome refers to the invisible barriers that prevent women from moving beyond a certain level on the career ladder. These barriers often arise as a result of gender roles, discriminatory policies, and prejudices. Glass ceiling syndrome creates multifaceted effects on female employees in the long term; these effects include not only professional success but also the psychological and physiological health of the individual [3]. Telogen effluvium, which is a problem closely related to stress, was found to be one of the physical symptoms that may be caused by glass ceiling syndrome in female employees.

Importantly, clinical interviews showed that in approximately 85% of participants, hair shedding started within 1–3 months after an identified workplace stressor related to the glass ceiling (e.g., promotion denial, perceived gender discrimination). This latency matches the well‐known 6–12‐week interval between a triggering event and the onset of TE [22, 23], reinforcing the plausibility of a causal link between occupational stress and hair loss in susceptible individuals.

As can be seen in this study, although a significant statistical relationship between glass ceiling syndrome and TE was found, it was also determined that women with glass ceiling syndrome have a high risk of developing telogen effluvium, and precautions should be taken. With this study, which is the first study on the relationship between glass ceiling syndrome and telogen effluvium, it is thought that women with glass ceiling syndrome, especially female employees working in the field of health, should receive help in coping with this chronic stress source, and this help may contribute to the treatment of diseases in female employees with TE. The importance of psychiatric support in the treatment of TE, especially in female employees with glass ceiling syndrome, is emphasized. In addition to this, the negative significant relationship between TE and occupational and educational status supports the idea that occupational and educational status may be a source of stress and supports the hypothesis that this stress level may cause TE. Glass ceiling syndrome was also found to be statistically significantly related to occupational status. It was observed that nurses and other health workers were exposed to glass ceiling syndrome more than doctors. In this case, in parallel with other studies in the literature, it is based on the belief that a higher education level is negatively related to glass ceiling syndrome.

## 5 Conclusion

Although it is seen that glass ceiling syndrome may cause TE in female healthcare workers, it is emphasized that this situation should not be neglected as a source of intense stress during treatment. At the same time, it was determined that the difference in the prevalence of glass ceiling syndrome between professions was closely related to the level of education. It is seen that nurses and other health workers are more prone to glass ceiling syndrome than doctors with higher education levels. It should not be overlooked that this situation is also directly related to TE.

## Conflicts of Interest

The authors declare no conflicts of interest.

## Data Availability

The data that support the findings of this study are available on request from the corresponding author. The data are not publicly available due to privacy or ethical restrictions.
